# Basic research and opportunities for translational advancement in the field of mammalian ∼12-hour ultradian chronobiology

**DOI:** 10.3389/fphys.2024.1497836

**Published:** 2024-11-20

**Authors:** William Dion, Bokai Zhu

**Affiliations:** ^1^ Aging Institute of UPMC, University of Pittsburgh School of Medicine, Pittsburgh, PA, United States; ^2^ Pittsburgh Liver Research Center, University of Pittsburgh, Pittsburgh, PA, United States; ^3^ Division of Endocrinology and Metabolism, Department of Medicine, University of Pittsburgh School of Medicine, Pittsburgh, PA, United States

**Keywords:** circadian rhythm, ultradian rhythm, proteostasis, NAFLD/MAFLD, nuclear speckles, XBP1s, unfolded protein response

## Abstract

Repetitive variations, such as oscillation, are ubiquitous in biology. In this mini review, we present a general summary of the ∼24 h circadian clock and provide a fundamental overview of another biological timekeeper that maintains ∼12 h oscillations. This ∼12 h oscillator is proposed to function independently of the circadian clock to regulate ultradian biological rhythms relevant to both protein homeostasis and liver health. Recent studies exploring these ∼12 h rhythms in humans are discussed, followed by our proposal that mammary gland physiology represents a promising area for further research. We conclude by highlighting potential translational applications in ∼12 h ultradian chronobiology.

## Introduction

Rhythms in organismal behavior, such as the sleep-wake cycle, and cellular-level oscillations are well-documented (McClung, 2007; Goldbeter, 2008). Among biological oscillations, circadian rhythms are the most well-characterized. These evolutionarily conserved timekeepers, found across various biological domains, regulate ∼24 hour (∼24 h) cycles that align closely with the Earth’s self-rotation (Pittendrigh, 1960; Loudon et al., 2000; Loudon, 2012). Besides circadian rhythms, there are other biological oscillations, such as infradian and ultradian rhythms, with periods longer or shorter than a day, respectively.

This mini review begins with an overview of the circadian clock, followed by a concise introduction to ∼12 hour (∼12 h) ultradian rhythms that play key roles in protein homeostasis and liver health. We then explore recent research on human ∼12 h ultradian chronobiology and present a rationale for studying ∼12 h gene expression rhythms in the mammary gland as a promising new research avenue. The comprehensive study of the circadian clock has already led to novel therapeutic strategies, advancing the field of chronotherapy (Festus et al., 2024). We propose that a deeper understanding of the molecular mechanisms governing ultradian rhythms, particularly the ∼12 h oscillator, could pave the way for new chronotherapies and reveal pharmacological targets to treat a variety of human diseases.

### The circadian clock is essential for organismal health

Circadian clocks can be entrained by environmental cues such as light, temperature, and food (entrainment), persist in their absence (free running), and maintain a ∼24 h period across a wide range of temperatures (temperature compensation) (Roenneberg and Merrow, 2005; Kidd et al., 2015; Mofatteh et al., 2021). The foundational study by Konopka and Benzer in 1971, titled “Clock Mutants of *Drosophila melanogaster*,” was the first to reveal how the molecular clock regulates circadian rhythms. They showed that mutations in the circadian gene *period* disrupt the circadian rhythms of eclosion and locomotion in fruit flies (Konopka and Benzer, 1971). In 1988, Ralph and Menaker demonstrated that a mutation in the *tau* gene alters the circadian rhythm of locomotor activity in the golden hamster, marking the first genetic link to circadian rhythms in a mammalian model (Ralph and Menaker, 1988).

The discovery of the *Clock* mutant in mice, which disrupts the circadian rhythm of wheel-running activity (Vitaterna et al., 1994), led to the identification of the CLOCK protein. Further research identified its binding partner, BMAL1, another core circadian clock transcription factor (Hogenesch et al., 1997; Ikeda and Nomura, 1997; Gekakis et al., 1998; Bunger et al., 2000). The BMAL1/CLOCK heterodimer regulates the transcription of two other circadian genes: *Period* and *Cryptochrome* (Gekakis et al., 1998; Jin et al., 1999; Kume et al., 1999). These genes encode the PER and CRY proteins, which together inhibit the transcriptional activity of CLOCK and BMAL1, thereby forming a negative feedback loop (Kume et al., 1999; Shearman et al., 2000; Partch et al., 2014). The transcriptional-translational feedback loop (TTFL) serves as the central mechanism driving circadian oscillations in output genes, which in turn regulate vital biological processes such as the sleep-wake and fast-feeding cycles, and many others (Roenneberg and Merrow, 2005; Takahashi, 2017; Rijo-Ferreira and Takahashi, 2019).

Our comprehensive understanding of circadian chronobiology has firmly established the connection between these ∼24 h rhythms and overall health. Research now shows that aligning feeding times (such as time-restricted feeding) with circadian rhythms can extend lifespan and promote healthy aging in mice (Acosta-Rodriguez et al., 2022). Conversely, disruptions in circadian rhythms have been causally linked to numerous negative health outcomes (Kettner et al., 2015; Cai et al., 2019; Fishbein et al., 2021; Huang et al., 2021; Huang et al., 2022; Sato and Sato, 2023; Van Drunen and Eckel-Mahan, 2023; Huang et al., 2024). These insights raise an important question: how do biological rhythms with periods other than ∼24 h influence mammalian health?

Our research group is particularly interested in ultradian rhythms, especially those cycling with a ∼12 h period, and their role in maintaining mammalian organismal health. Exploring how these faster biological oscillations influence health could reveal new opportunities for therapeutic interventions, much like the advancements made through circadian research. In the following sections, we will delve into recent discoveries on these fascinating ∼12 h ultradian rhythms.

### The ∼12 h oscillator is essential for maintaining (ER) proteostasis

Endoplasmic reticulum (ER) protein homeostasis (proteostasis) is vital for maintaining a healthy secreted proteome (Plate and Wiseman, 2017). Newly synthesized proteins enter the ER, where they are properly folded and assembled for secretion. The unfolded protein response (UPR) plays a critical role in managing ER proteostasis. When misfolded proteins accumulate in the ER lumen, the UPR is activated through three ER membrane proteins: ATF6, IRE1α, and PERK. These proteins can sense ER stress and quickly initiate signaling cascades to either restore proteostasis or, if the stress is too severe, trigger apoptosis (Walter and Ron, 2011; Hetz et al., 2015).

Of these, the IRE1α branch of the UPR is the most evolutionarily conserved (Karagöz et al., 2017). Under normal conditions, the ER chaperone BiP binds to IRE1α in the ER lumen, keeping it inactive as a monomer. When unfolded proteins accumulate, they compete with IRE1α for BiP binding, freeing IRE1α and allowing it to activate through oligomerization and autophosphorylation. This activation enables the endoribonuclease domains of IRE1α to splice *Xbp1* mRNA in a non-canonical manner, producing the spliced form *Xbp1s* by releasing a 26-nucleotide intron. *Xbp1s* mRNA is translated into the ∼50 kD transcription factor XBP1s that moves to the nucleus and initiates a transcriptional response aimed at restoring proteostasis (Ron and Walter, 2007; Karagöz et al., 2019). Importantly, XBP1s also plays a central role in regulating the ∼12 h oscillator, as demonstrated below.

Studies by Hughes et al. (2009) and Cretenet et al. (2010) were among the first to link ∼12 h rhythms to ER proteostasis (Hughes et al., 2009; Cretenet et al., 2010). Hughes et al. (2009) showed ∼12 h rhythmic expression of selective ER proteostasis genes including *BiP* in the liver of mice that are fed *ad libitum* under constant darkness conditions, while Cretenet et al. (2010) further demonstrated ∼12 h oscillations in the IRE1α branch of the UPR in mouse liver, including ∼12 h rhythms of phosphorylated IRE1α and nuclear XBP1s levels (Cretenet et al., 2010). Their work also showed that the loss of *Cry1* and *Cry2* genes disrupted this ∼12 h rhythmicity. By contrast, later studies found that hundreds of ∼12 h hepatic transcripts including *Bip* and *Xbp1s* persisted even without BMAL1, the central circadian clock regulator (Yang et al., 2016; Zhu et al., 2017; Zhu and Liu, 2023). This discrepancy in how disrupting different components of the circadian clock affects ∼12 h rhythms may stem from the non-circadian clock-regulating functions of the CRY1/CRY2 proteins (Wong et al., 2022; Zhu and Liu, 2023).

Zhu et al. (2017) subsequently identified XBP1s as a key transcriptional regulator of ∼12 h rhythms of gene expression via directly binding to the promoter regions of many ER proteostasis genes, with prominent examples such as *BiP*, *Eif2ak3* and *Sec23b* (Zhu et al., 2017; Zhu et al., 2018). *Eif2ak3* encodes PERK, one of the sensors of ER stress that triggers the integrated stress response to attenuate global translation (Pakos-Zebrucka et al., 2016; Costa-Mattioli and Walter, 2020). *Sec23b* plays a role in exporting proteins from the ER for secretion (Tao et al., 2012). By performing high temporal resolution hepatic transcriptome profiling in both wild-type and XBP1 liver-specific knockout mice, Pan et al. (2020) demonstrated that XBP1s liver-specific ablation minimally affects the hepatic circadian transcriptome but greatly disrupts the ∼12 h oscillating gene program (Pan et al., 2020). Hepatic XBP1s ChIP-Seq revealed direct ∼12 h rhythmic XBP1s chromatin recruitment to the promoter regions of hundreds of genes (Pan et al., 2020). XBP1s-dependent hepatic ∼12 h cycling genes are strongly enriched in the proteostasis pathways, including ribosome biogenesis, protein processing in the ER and Golgi, protein folding, and protein export (Pan et al., 2020; Meng et al., 2020). Lastly, XBP1s-dependent cell-autonomous ∼12 h oscillations of proteostasis gene expression were further identified in mouse embryonic fibroblasts (Zhu et al., 2017; Pan et al., 2020).

Together, these studies indicate that ∼12 h ultradian rhythms operate through mechanisms distinct from circadian timekeeping and instead involve a dedicated “∼12 h oscillator”. These results further establish XBP1s as a central transcriptional regulator of the ∼12 h oscillator, playing a critical role in proteostasis. However, it is premature to conclude that the ∼12 h oscillator operates entirely independently of the circadian clock. The circadian clock regulates feeding behavior and cellular metabolism (Vollmers et al., 2009; Page et al., 2020; Schrader et al., 2024). Since metabolic cues are known to entrain the ∼12 h oscillator (Zhu et al., 2017), disruptions in circadian rhythms could indirectly influence ∼12 h ultradian rhythms via altered behaviors and metabolism.

#### Nuclear speckles are integral components of the ∼12 h oscillator and essential for (ER) proteostasis

In mice, besides proteostasis genes, mRNA metabolism genes also exhibit ∼12 h oscillations across various tissues (Zhu, 2020; Zhu and Liu, 2023), but the mechanism linking mRNA metabolism with proteostasis dynamics remains unclear. Our research group aims to uncover this connection by studying nuclear speckles—biomolecular condensates that regulate aspects of mRNA metabolism, including transcription, mRNA splicing, and RNA export (Spector and Lamond, 2011; Liao and Regev, 2021; Hirose et al., 2023; Bhat et al., 2024).

Nuclear speckles contain RNA-protein complexes called spliceosomes that are essential for RNA processing (Girard et al., 2012). Notably, the Gene Ontology (GO) term “spliceosome” is just as enriched in the XBP1s-dependent hepatic ∼12 h transcriptome as GO terms related to proteostasis (Pan et al., 2020). These speckles are believed to form via liquid-liquid phase separation (LLPS), where proteins rich in intrinsically disordered regions, such as SRRM2 and SON, create a scaffold that facilitates the assembly of other proteins (including splicing factors) and RNAs (such as the long non-coding RNA *Malat1*), forming a heterogeneous condensate with wide-ranging viscoelastic properties (Sharma et al., 2010; Sharma et al., 2011; Fei et al., 2017; Ilik et al., 2020; Ilik and Aktas, 2022). Components of nuclear speckles continuously exchange between the dense phase (the speckle itself) and the dilute phase (the surrounding nucleoplasm), resulting in irregular shapes and dynamic morphologies (Banani et al., 2017; Ilik and Aktas, 2022; Hirose et al., 2023). The proximity of nuclear speckles to genes influences transcription, with closer speckles often associated with higher transcriptional activity (Kim et al., 2020; Alexander et al., 2021; Bhat et al., 2024). This indicates that the shape and size of nuclear speckles can influence the cellular transcriptome, as larger speckles with greater surface area are likely to interact with more chromatin, potentially enhancing the expression of nearby genes (Dion et al., 2022).

**FIGURE 1:**
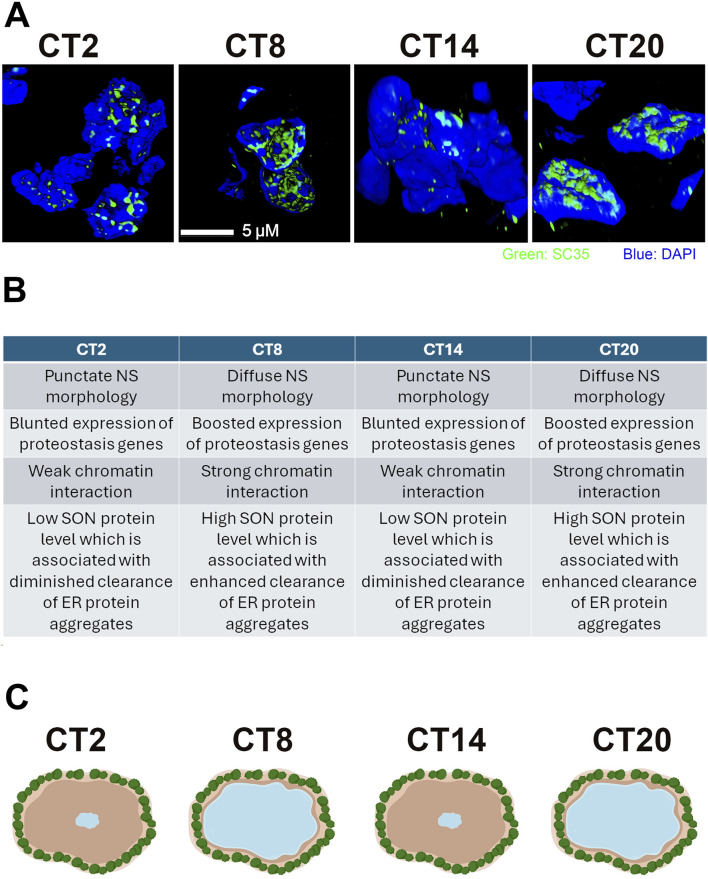
Ultradian biological rhythms of nuclear speckle liquid-liquid phase separation dynamics and proteostasis. **(A)** Nuclear speckle morphology (indicated by SC35 signal) in mouse liver at different timepoints. Normal nuclear speckle morphologies alternate between punctate (CT2 and CT14) and diffuse (CT8 and CT20). Panel taken from Figure 1A of Dion et al., 2022, ^©^ The Authors, some rights reserved; exclusive licensee AAAS. Distributed under a Creative Commons Attribution License 4.0 (CC BY) https://creativecommons.org/licenses/by/4.0/. **(B)** Characteristics associated with different nuclear speckle morphologies (nuclear speckle (NS), liquid-liquid phase separation (LLPS)). **(C)** Our lake analogy shows full and drying lakes representing normally occurring nuclear speckle morphologies and green shrubs which represent chromatin. The changes in the water’s distance from the green shrubs could be understood as how nuclear speckles' proximity to chromatin normally changes over time. Image created with BioRender.com.

Our group has linked the LLPS dynamics of nuclear speckles to the expression of proteostasis genes by uncovering an XBP1s-SON regulatory axis. This axis controls ∼12 h rhythms in both nuclear speckle morphology (Figure 1A) and their interactions with chromatin (Dion et al., 2022). The expression level of SON, a key scaffolding protein in nuclear speckles, significantly influences their LLPS dynamics, which in turn affects the transcription of proteostasis genes (Dion et al., 2022). Elevated SON levels increase the diffuseness and surface area of nuclear speckles, enhancing their interaction with chromatin, amplifying the expression of proteostasis genes (including *Xbp1*), and reducing protein aggregation (Dion et al., 2022). Conversely, reducing SON expression has the opposite effect, leading to smaller speckles with decreased chromatin interaction and lower proteostasis gene expression (Dion et al., 2022). Notably, *Son* is a direct transcriptional target of XBP1s, establishing a direct link between nuclear speckle dynamics and the transcriptional regulation of proteostasis (Dion et al., 2022).

Under physiological conditions, SON expression, nuclear speckle LLPS dynamics, chromatin interactions, and proteostasis gene expression all exhibit XBP1s-dependent ∼12 h rhythms (Figures 1A,B) (Dion et al., 2022). These insights led us to develop the “lake analogy”: when SON levels are high, nuclear speckles become large and diffuse, akin to a full lake, with strong chromatin interactions. In contrast, when SON levels are low, the speckles are smaller and more spherical, resembling a drying lake, with weaker chromatin interactions (Figure 1C) (Dion et al., 2022). Our exploration of ∼12 h ultradian chronobiology has deepened our understanding of the molecular mechanisms underlying proteostasis and identified nuclear speckles as a new therapeutic target for proteinopathies (Dion et al., 2024).

### The ∼12 h oscillator regulates liver health via lipid remodeling

Liver disease is a growing problem affecting diverse populations (Loomba and Sanyal, 2013; Byrne and Targher, 2015; Kardashian et al., 2023; Younossi et al., 2023). Non-alcoholic fatty liver disease (NAFLD) (or “metabolic dysfunction-associated fatty liver disease” (MAFLD) (Chen et al., 2024)) is associated with dysfunctional ER proteostasis (Flessa et al., 2022). While the loss of either UPR or ER quality control components results in hepatic steatosis (also known as “fatty liver”) in mice, maintaining or activating ER quality control mechanism protects against NAFLD (Rutkowski et al., 2008; Yamamoto et al., 2010). For instance, IRE1α maintains lipid balance during ER stress (Zhang et al., 2011) and XBP1s reduces the production of lipids in the livers of both obese and insulin-resistant mice (Herrema et al., 2016). XBP1s-selective pharmacological activation of IRE1α also improves liver function in obese mice (Madhavan et al., 2022). The importance of the ∼12 h oscillator’s regulator XBP1s to hepatic function suggests a link between ∼12 h ultradian rhythms and liver health.

In addition to regulating mRNA metabolism and proteostasis, XBP1s also plays key roles in lipid metabolism (Moncan et al., 2021). Recent studies have shown that activating XBP1s and other UPR pathways can protect against hepatic steatosis by modulating membrane lipid composition (Rutkowski et al., 2008; Yamamoto et al., 2010; Zhang et al., 2011; Herrema et al., 2016). For instance, during diet-induced ER stress, activation of the *Lysophosphatidylcholine Acyltransferase 3* (*Lpcat3*) gene, which promotes the incorporation of polyunsaturated fatty acids into ER membrane phospholipids, helps maintain ER membrane fluidity, reducing both hepatic inflammation and ER stress (Rong et al., 2013; Zhu et al., 2017). Notably, *Lpcat3* mRNA and levels of 2-Lysophosphatidylcholine species (LPCAT3 catalyzes the conjugation of 2-Lysophosphatidylcholine with unsaturated Acyl-CoA to form phosphatidylcholine) exhibit strong ∼12 h rhythms in the mouse liver, along with rhythmic expression of fatty acid-modifying enzymes like *Scd1* and *Elovl6* (Zhu et al., 2017; Zhu et al., 2018; Meng et al., 2020). These rhythmic changes in lipid composition impact the fluidity of cellular membranes, affecting signal transduction across lipid bilayers and potentially influencing systemic metabolism—a connection that remains to be fully explored. In mice with liver-specific XBP1 deletion, the ∼12 h rhythm of *Lpcat3* expression is disrupted, leading to lower levels of polyunsaturated phospholipids, reduced membrane fluidity, and impaired lipid metabolism (Meng et al., 2020). This disruption accelerates the development of NAFLD and liver aging, while also contributing to glucose intolerance and hyperinsulinemia (Meng et al., 2020).

In a follow-up study, Meng et al. (2022) characterized SRC-3 (*Ncoa3*) as a transcriptional co-activator of XBP1s essential for hepatic ∼12 h rhythms of gene expression and proper metabolic function (Meng et al., 2022). Considering the loss of ∼12 h hepatic rhythms of gene expression preceded the manifestation of steatosis (Meng et al., 2020), disruption of the hepatic ∼12 h oscillator is suggested to drive, rather than be a consequence of, NAFLD. Chronobiological therapies that maintain ∼12 h ultradian rhythmicity could prevent or slow the progression of NAFLD. One future research direction could be administering the XBP1s-selective IRE1α activating compound IXA4 (Grandjean et al., 2020; Madhavan et al., 2022) at regular intervals to possibly synchronize/reinforce the ∼12 h oscillator. This approach could be applied to different mouse models of NAFLD to see if pharmacologically boosting the ∼12 h oscillator could slow or prevent liver disease progression.

### ∼12 h rhythms exist in humans

Previous research has identified ∼12 h oscillations in human physiological metrics, suggesting the existence of a ∼12 h oscillator in humans (Broughton and Mullington, 1992; Wan et al., 1992; Hayashi et al., 2002; Otsuka et al., 2016; Otsuka et al., 2022; Otsuka et al., 2023a; Otsuka et al., 2023b). As previously discussed, the ∼12 h oscillator plays a crucial role in regulating proteostasis in mice, and disruptions in proteostasis are also strongly linked to human neurodegenerative diseases such as Alzheimer’s, Parkinson’s, and Huntington’s, as well as psychiatric disorders like schizophrenia (SZN) (Hetz and Saxena, 2017). For example, altered expression of XBP1s in the brain was observed in Alzheimer’s and Huntington’s diseases (Hetz and Mollereau, 2014), while dysfunction of the IRE1α component of the UPR has been linked to SZN (Kim et al., 2021).

To explore whether ∼12 h rhythms exist in the human brain and their potential connection to psychiatric disorders, Scott et al. (2023) conducted a *post hoc* analysis of RNA-seq data from human brain samples, using time-of-death as a proxy for circadian time. The analysis included both control subjects and individuals with SZN (Scott et al., 2023). In the dorsolateral prefrontal cortex—a region critical for cognitive function—Scott et al. (2023) identified ∼12 h rhythms of gene expression in control subjects. These rhythms peaked at sleep/wake transitions (around 9 AM and 9 PM) and at static times (around 3 AM and 3 PM) (Scott et al., 2023). Intriguingly, in subjects with SZN, genes associated with the UPR and neuronal structural maintenance lost their ∼12 h rhythmic expressions (Scott et al., 2023). Additionally, genes involved in mitochondrial function and protein translation, which normally peak at sleep/wake transitions in control subjects, peak at static times in SZN subjects (Scott et al., 2023). These findings suggest that pharmacological realignment of ∼12 h gene expression rhythms might help alleviate some symptoms of schizophrenia. This approach aligns with existing strategies that target the circadian clock as a therapeutic option for circadian disruptions (Rasmussen et al., 2022).

In a separate study, Zhu et al. (2024) provided direct evidence of ∼12 h ultradian rhythms in humans through prospective temporal transcriptome profiling of peripheral white blood cells from three healthy male subjects (Zhu et al., 2024). This study identified robust ∼12 h transcriptional rhythms, particularly those implicated in RNA and protein metabolism, with striking homology to the circatidal gene programs previously found in marine species like Cnidarians (Zhu et al., 2024). In addition, Zhu et al. (2024). uncovered ∼12 h rhythms of intron retention in genes involved in MHC class I antigen presentation, which were synchronized with mRNA splicing gene expression in each individual (Zhu et al., 2024). These findings suggest that human ∼12 h biological rhythms have a primordial evolutionary origin and may have significant implications for human health and disease beyond neurological disorders and metabolic syndromes.

### Mammary gland physiology as a future direction

The synthesis and secretory demands of lactation are associated with an abundance of Golgi and ER in alveolar epithelial cells (Anderson et al., 2007; Hannan et al., 2023). *Xbp1* gene expression increases in the pregnant murine mammary gland (Tsuchiya et al., 2017), and knockout of *Xbp1* in the mammary epithelium caused ER stress during lactation and impeded milk production (Hasegawa et al., 2015; Davis et al., 2016). The transcriptional co-activator SRC-3 is also essential for proper mammary gland development (Xu et al., 2000). These studies show that previously identified aspects of the ∼12 h oscillator are relevant to mammary gland physiology.

**FIGURE 2:**
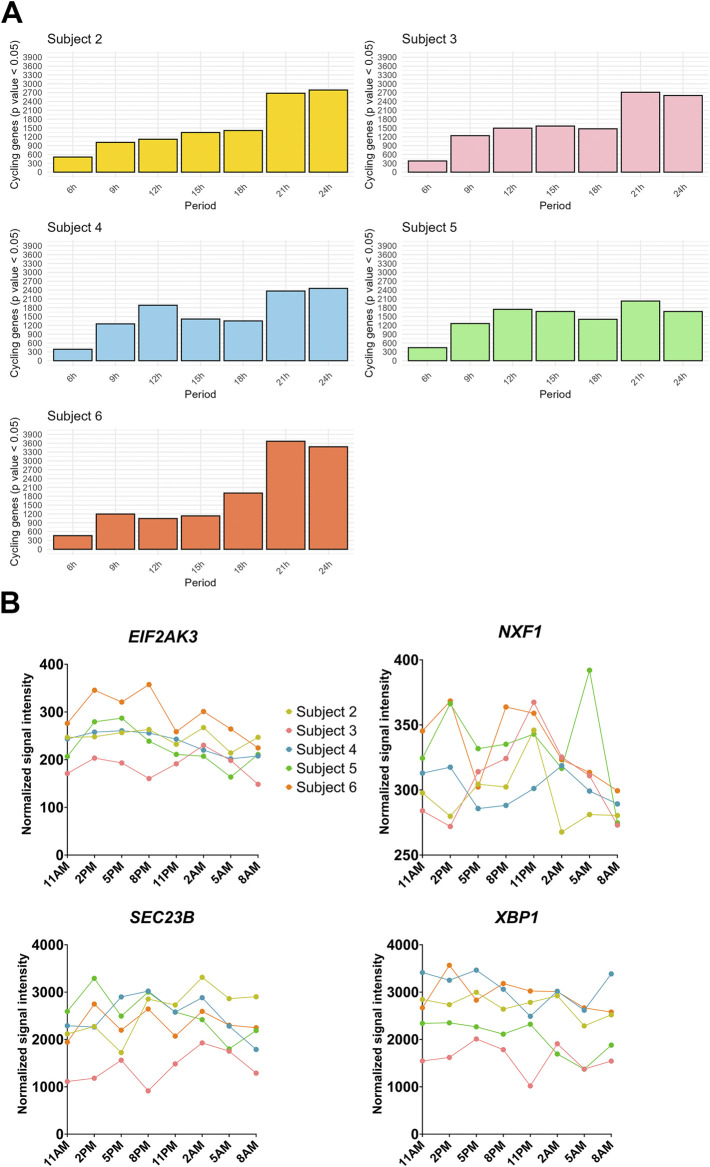
*Post hoc* analysis of the human milk fat globule temporal transcriptome. Data from the published study are available through the NCBI Gene Expression Omnibus, identifier GSE12669 (Maningat et al., 2009). **(A)** Total cycling genes with *p* values less than 0.05 for corresponding periods as determined with RAIN (Thaben and Westermark, 2014). **(B)** Temporal expression profiles of individual genes previously shown to have ultradian rhythms (Zhu et al., 2017; Pan et al., 2020).

Maningat et al. (2009) completed a temporal analysis of human milk fat globule (hMFG) gene expression to study the cycling transcriptome of human mammary epithelial cells (Maningat et al., 2009). They uncovered a circadian transcriptional program in the hMFG which prompted our *post hoc* analysis of their published gene expression data. We used RAIN (Thaben and Westermark, 2014) to test for ultradian oscillations of gene expression and uncovered a distribution of cycling genes with ultradian and circadian periods among the participants (Maningat et al., 2009) (Figure 2). Based on these findings, we propose that there is strong justification for a study profiling the temporal transcriptome in murine mammary glands, both with and without functional XBP1s activity. Such research could help identify chronotherapeutic targets that address barriers to healthy lactation, ultimately benefiting mothers and their infants (Rollins et al., 2016; Wang and Scherer, 2019).

### Closing remarks

Our understanding of ∼12 h biological rhythms in humans, though largely descriptive at this point, suggests translational studies are an appropriate future direction. Pharmacological adjustment of circadian rhythms is a proposed therapy to address the disruption of the circadian clock caused by jetlag (Ruan et al., 2021). This suggests that manipulating other biological timekeepers may also benefit human health. The loss of ∼12 h rhythmicity preceding NAFLD progression (Meng et al., 2020) and the misalignment of ∼12 h rhythms in the dorsolateral prefrontal cortex of individuals with SZN (Scott et al., 2023) are two previously discussed examples in which synchronizing or realigning ∼12 h ultradian rhythms could prove as effective therapies. Perhaps inducing low levels of ER stress—which synchronizes the ∼12 h oscillator (Zhu et al., 2017)—to reset ultradian biological rhythms could be an effective chronotherapy to slow NAFLD progression or address some symptoms of SZN. Furthermore, the development of compounds that specifically activate the UPR—such as IXA4 mentioned previously (Grandjean et al., 2020; Madhavan et al., 2022)—could prove to be convenient therapies to manipulate ultradian rhythms.

Despite significant progress in the field of ultradian chronobiology, there is still much more to be learned. We encourage others to uncover more of the molecular clockwork regulating ∼12 h rhythms. Such discoveries promise to benefit human health, given the recent establishment of ∼12 h transcriptional programs among different human tissues. Our understanding of the ∼12 h oscillator is biased toward males in both mice and humans and present studies focus heavily on the liver. We firmly believe that future studies across both sexes and of different tissue types are essential to understanding the full translatable impact of the ∼12 h oscillator on human health.
